# Single Test‐Based Diagnosis and Subtyping of Pulmonary Hypertension Caused by Fibrosing Mediastinitis Using Plasma Metabolic Analysis

**DOI:** 10.1002/advs.202416454

**Published:** 2025-03-06

**Authors:** Yating Zhao, Chunmeng Ding, Hongling Su, Aqian Wang, Aiping Tang, Hongfan Zhao, Ya Ma, Min Zhang, Wanshan Liu, Ruimin Wang, Ziyue Zhang, Shouzhi Yang, Dingyitai Liang, Yida Huang, Kun Qian, Lin Huang, Qihua Fu, Yunshan Cao

**Affiliations:** ^1^ Heart, Lung and Vessels Center Sichuan Provincial People's Hospital University of Electronic Science and Technology of China Chengdu Sichuan 610072 China; ^2^ School of Medicine Jiangsu University Zhenjiang 212000 China; ^3^ Department of Cardiology Pulmonary Vascular Disease Center (PVDC) Gansu Provincial Hospital Lanzhou 730000 China; ^4^ State Key Laboratory of Systems Medicine for Cancer School of Biomedical Engineering Institute of Medical Robotics and Shanghai Academy of Experimental Medicine Shanghai Jiao Tong University Shanghai 200030 China; ^5^ The First Clinical Medical College of Gansu University of Chinese Medicine (Gansu Provincial Hospital) Lanzhou 730000 China; ^6^ The First Clinical Medical School Lanzhou University Lanzhou 730000 China; ^7^ Clinical Research Center Sichuan Provincial People's Hospital University of Electronic Science and Technology of China Chengdu Sichuan 610072 China; ^8^ Department of Clinical Laboratory Medicine Shanghai Chest Hospital, Shanghai Jiao Tong University Institute of Thoracic Oncology Shanghai Chest Hospital Shanghai Jiao Tong University School of Medicine Shanghai 200030 China; ^9^ Center for Medical Genetics and Rare Diseases Sichuan Provincial People's Hospital University of Electronic Science and Technology of China Chengdu Sichuan 610072 China

**Keywords:** diagnosis, laser desorption/ionization mass spectrometry, metabolic analysis, pulmonary hypertension caused by fibrosing mediastinitis, subtyping

## Abstract

Pulmonary hypertension (PH) often leads to poor survival outcomes and encompasses diverse subtypes with distinct underlying causes. Specifically, PH resulting from fibrosing mediastinitis (FM‐PH) presents significant diagnostic challenges due to nonspecific symptoms and overlap of clinical characterization with other PH subtypes, leading to frequent misdiagnosis and delayed treatment. Moreover, the complex diagnostic procedures impose a significant burden on FM‐PH patients, many of whom already experience mobility difficulties. This study represents a single test‐based diagnosis of FM‐PH, using the plasma metabolites obtained through ferric particle‐enhanced laser desorption/ionization mass spectrometry analysis. Distinct metabolic alterations in FM‐PH are identified compared to healthy controls and other PH subtypes, achieving an area under the curve (AUC) of 0.987 for FM‐PH diagnosis and 0.728 for differentiating FM‐PH from other subtypes. By addressing existing gaps in diagnostic strategies, this research highlights the potential of metabolic analysis in elucidating the metabolic landscape of PH.

## Graphical abstract

Pulmonary hypertension caused by fibrosing mediastinitis has a five‐year survival rate of just 56%. Current diagnostic approaches are complex for patients with mobility limitations and can lead to misdiagnoses due to their nonspecific clinical features. This work presents a straightforward and precise strategy based on metabolic analysis—a single test with an accuracy of 93.6%—to complement existing diagnostic modalities.

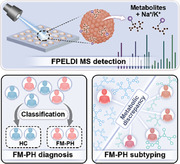

## 1 Introduction

Pulmonary hypertension (PH) is clinically defined as organ dysfunction with an elevated mean pulmonary artery pressure (mPAP) > 20 mmHg at rest.^[^ ^1^ ^]^ The prevalence of non‐specific clinical conditions (dyspnoea, chest pain, hemoptysis, etc.) among patients with PH leads to delayed diagnosis and treatment, which further contributes to poor prognosis (overall mean survival < 5 years).^[^ ^1^ ^]^ PH encompasses a heterogeneous spectrum of diseases, and treatment strategies vary significantly across the five clinical classification subtypes (defined by the World Health Organization (WHO)).^[^ ^2^ ^]^ Especially, PH resulting from fibrosing mediastinitis (FM‐PH) is classified as a subtype within the WHO group 5 PH.^[^ ^3^ ^]^ The low prevalence (less than 0.02%) and nonspecific clinical features of FM‐PH contribute to its frequent misdiagnosis and underdiagnosis, hindering appropriate treatment guidance.^[^ ^1^, ^4^ ^]^ When left untreated, this subtype frequently leads to fatal obstruction of the pulmonary vasculature and airways, resulting in a five‐year survival rate of only 56%.^[^ ^4^ ^]^ Therefore, developing diagnostic and subtyping strategies is essential to enhance the precision management of FM‐PH.

The often‐insidious nature of FM‐PH, coupled with the necessity for extensive investigations, makes definitive diagnosis a complex challenge, particularly in resource‐limited settings.^[^ ^4^ ^]^ Right heart catheterization (RHC) is the gold standard for PH diagnosis; however, it carries invasive risks that should not be overlooked. Additionally, the application of RHC in remote and impoverished areas is limited by transportation and cost challenges. Due to these risks and the limited availability of RHC, echocardiography has become the primary tool for preliminary screening and diagnosis of PH. Following echocardiographic evaluation, high‐risk patients still need confirmation through RHC and etiology identification via ventilation/perfusion (V/Q), high‐resolution CT of the lungs, CT pulmonary angiography, etc.^[^ ^5^ ^]^

Compared to existing diagnostic methods which are time‐consuming and professionally dependent, blood tests incorporating “deep phenotyping” information of biomarkers are given high attention in PH diagnosis and subtyping.^[^ ^5^, ^6^ ^]^ In particular, metabolites have become increasingly important in interpreting the pathology of PH. Initially, metabolic heterogeneity attracted attention in pulmonary arterial hypertension (PAH, WHO group 1 PH), where several metabolites from lung tissues exhibited altered metabolic pathways that distinguished PH patients from healthy controls (HC).^[^ ^7^ ^]^ Notably, research on breath metabolic biomarkers has emerged, indicating their potential utility in diagnosing PAH.^[^ ^8^ ^]^ Additionally, specific plasma metabolic biomarkers became increasingly significant for assessing the disease state and prognosis in PAH patients, demonstrating the clinical applicability of metabolic heterogeneity in PAH.^[^ ^9^ ^]^ Furthermore, such investigations also examined metabolic profiles in other subtypes of PH. An untargeted metabolic analysis identified a panel of metabolic profiles that distinguish PH related to chronic thromboembolic disease (CTEPH) from both HC and idiopathic PAH, highlighting the potential application of metabolic biomarkers in PH subtyping.^[^ ^10^ ^]^ Despite these advancements, significant gaps remain in the studies of metabolic perturbation specifically related to FM‐PH. Additionally, the analysis of metabolic differences among various PH subtypes remains unexplored.

This study used a recently developed ferric particle‐enhanced laser desorption/ionization mass spectrometry (FPELDI MS) analysis to obtain metabolic profiles of FM‐PH, HC, and other PH subtypes. The optimized metabolic model achieved an AUC of 0.987 for diagnosing FM‐PH. Additionally, the differential metabolites between FM‐PH and other PH subtypes yielded an AUC of 0.728, enhancing a deep understanding of the metabolic alterations associated with various PH subtypes. Our findings underscore the potential for integrating metabolic profiling into clinical practice, facilitating early diagnosis and accurate therapeutic approaches for patients with PH.

## 2 Results

### 2.1 Sample Characteristics and Study Design

A retrospective collection of plasma samples from 290 patients subsequently diagnosed with PH was selected for this study. 150 HC participants were collected during routine physical examination as a control group. Four types of PH (defined by the WHO)^[^ ^11^ ^]^ were incorporated with the following distribution: PAH (WHO group 1, *n* = 78, age 44.4 ± 1.8 years, 76.9% females), PH associated with hypoxic (HPH, WHO group 3, *n* = 27, age 60.7 ± 2.2 years, 55.6% females), PH related to chronic thromboembolic disease and pulmonary Takayasu arteritis (CTEPH/PTA, WHO group 4, *n* = 36, age 54.6 ± 2.0 years, 75.0% females), and PH caused by fibrosing mediastinitis (FM‐PH, *n* = 149, age 66.7 ± 0.6 years, 60.4% females).

The inclusion‐exclusion cascade is summarized in Figure S1 (Supporting Information). The age and gender information for each participant was collected to assemble the training cohort and test cohort, allowing for the development and validation of the diagnostic model. In addition, the results of RHC diagnosis, echocardiography, WHO functional class (WHO‐FC), six minutes walk distance (6MWD), and *N*‐terminal pro‐brain natriuretic peptide (NT‐proBNP) of patients were obtained to describe the disease cohort. Table S1 (Supporting Information) summarized the demographic information of the participants, indicating that PH disorder disproportionately affects women. Additionally, distinct age preferences were observed among patients with certain subtypes of PH. For instance, elderly patients with FM‐PH are more prevalent in China, which is associated with the disease triggers (e.g., *mycobacterium tuberculosis* infection).^[^ ^3^ ^]^ Correspondingly, FM‐PH patients commonly exhibit limitations in movement, as indicated by poorer results of the 6MWD, emphasizing the importance of a convenient diagnostic approach and streamlined diagnostic process.

**Scheme 1:**
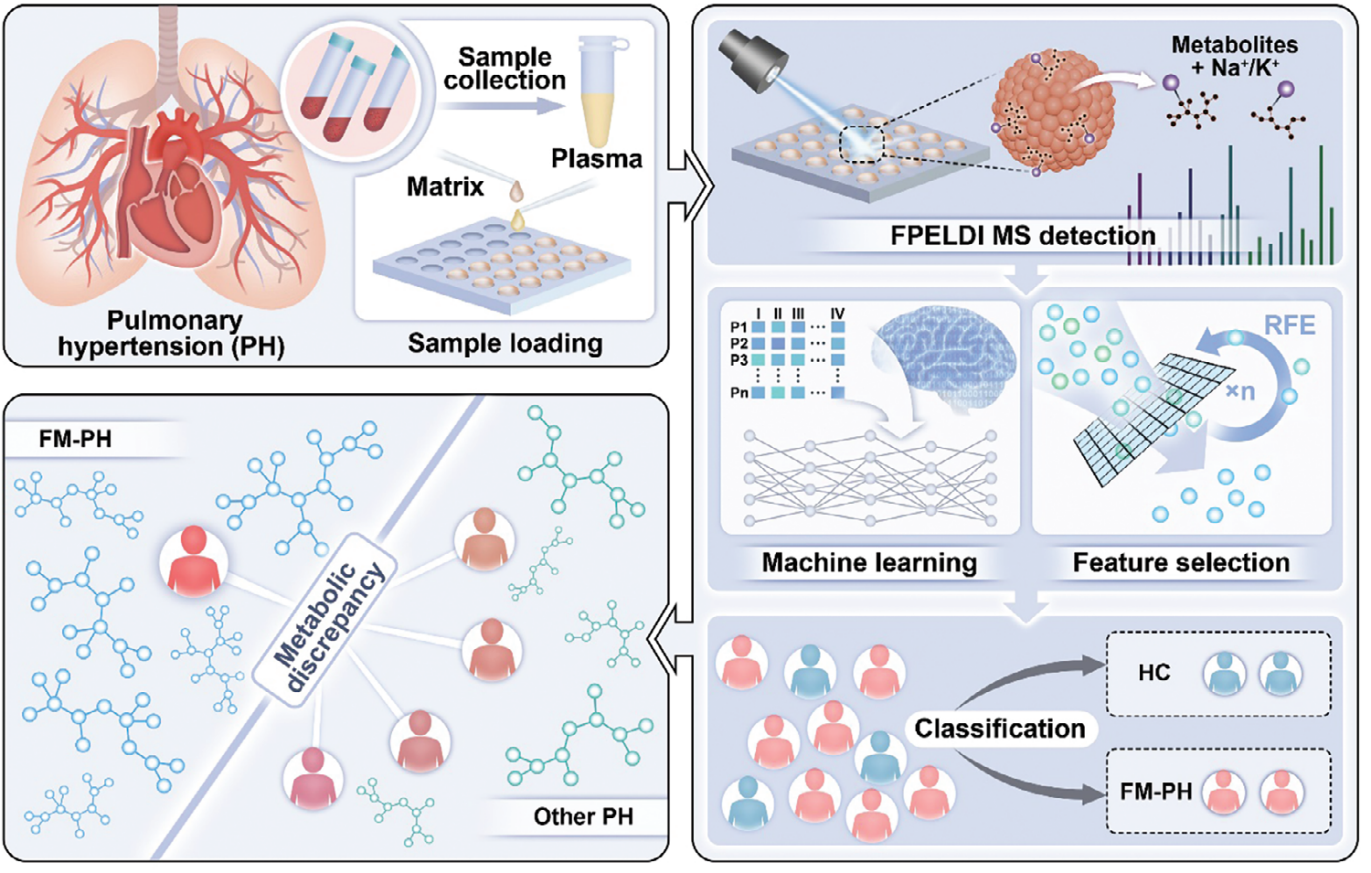
Overview of the study design. Metabolic profiles of plasma were obtained by ferric particle‐enhanced laser desorption/ionization mass spectrometry (FPELDI MS) and analyzed by machine learning. The system output includes the selection of representative altered metabolites and predictions regarding the presence of pulmonary hypertension resulting from fibrosing mediastinitis (FM‐PH) and healthy controls (HC).

We performed metabolic analysis on each sample using FPELDI MS for data acquisition (**Scheme** **1**). After deproteinization pretreatment, plasma samples were loaded onto the polished target plate (384 dots, Figure S2A, Supporting Information) and allowed to dry naturally for subsequent MS analysis. Ferric oxide particles served as effective matrices to assist MS analysis. We obtained the data after preprocessing steps, including metabolite extraction and baseline correction. Subsequently, we conducted several comparisons to identify differences between PH and HC samples.

### 2.2 Metabolic Landscape of FM‐PH

**Figure 1:**
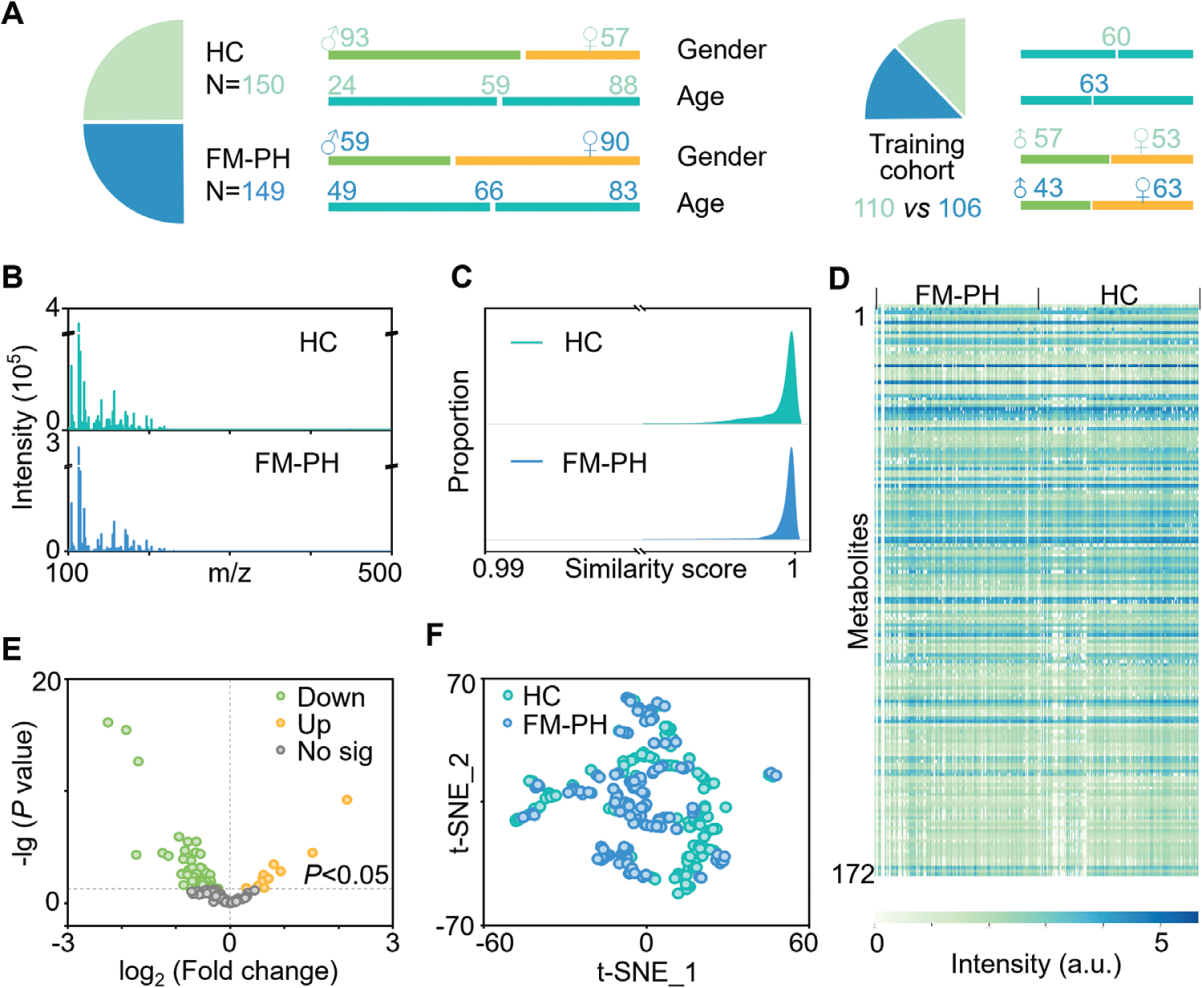
Metabolic landscape of FM‐PH. A) Demographics of the HC and FM‐PH participants. Age and gender were matched with no significant differences (*p* > 0.05) in the training cohort. B) Representative mass spectra and C) cosine similarity scores of the FPELDI MS analysis within the HC group and FM‐PH group individually. D) 172 metabolites were obtained from the metabolic analysis of all samples. E) Volcano diagram of HC and FM‐PH comparison. Eighty‐two metabolites were significantly different between the two groups. F) T‐distributed stochastic neighbor embedding result exhibited a minor degree of separation between HC and FM‐PH groups.

The experiment began by dividing the FM‐PH and HC samples into training and test cohorts (**Figure** **1**A). Following random assignment, we adjusted the composition to obtain a training cohort with no significant difference (FM‐PH vs HC) in age (*p* = 0.053) and gender (*p* = 0.097). This training cohort included 110 HC participants (average age 60 years, 48% female) and 106 FM‐PH patients (average age 63 years, 59% female). Excluding the effects of gender and age on disease characterization was important for constructing the model. The test cohort (FM‐PH:HC = 43:40) had a slightly higher proportion of female patients.

Figure 1B exhibited representative FPELDI MS spectra of metabolites from FM‐PH and HC groups, displaying subtle variations in signal intensities. Since the ferric particles had uniform particle size (197.5 ± 13.0 nm, Figure S2B,C, Supporting Information) and crystallization (surface arithmetic mean deviation = 0.335 µm, Figure S3A, Supporting Information), the uniform hotspot arrangement facilitated the acquisition of relatively stable metabolite signals. The coefficient of variation distribution of metabolite intensities from ten independent replicates across three plasma samples was 12.9%–14.8% (Figure S3B, Supporting Information), demonstrating the high reproducibility of FPELDI MS. We calculated the cosine similarity of detection results for the within‐group samples. Both groups had similarity scores above 0.9, indicating respective metabolic similarity within the groups (Figure 1C).

Different metabolite constituents were investigated to confirm the major difference between HC and FM‐PH patients. A total of 172 metabolites were extracted from the plasma metabolic analysis, providing an overview of the metabolic characterization (Figure 1D). The plasma metabolic fingerprints illustrated the prospective discrepancy between HC and FM‐PH groups in Figure 1E. Among the 172 metabolites, we identified 11 upregulated and 71 downregulated (*p* < 0.05, fold change < 1) metabolites in FM‐PH patients compared to the HC group. T‐distributed stochastic neighbor embedding analysis indicated a trend of metabolic remodeling in FM‐PH patients (Figure 1F). More effective strategies are needed to highlight the distinct features of FM‐PH and facilitate more accessible diagnostic approaches.

### 2.3 FM‐PH Diagnostic Model Derivation and Validation

**Figure 2:**
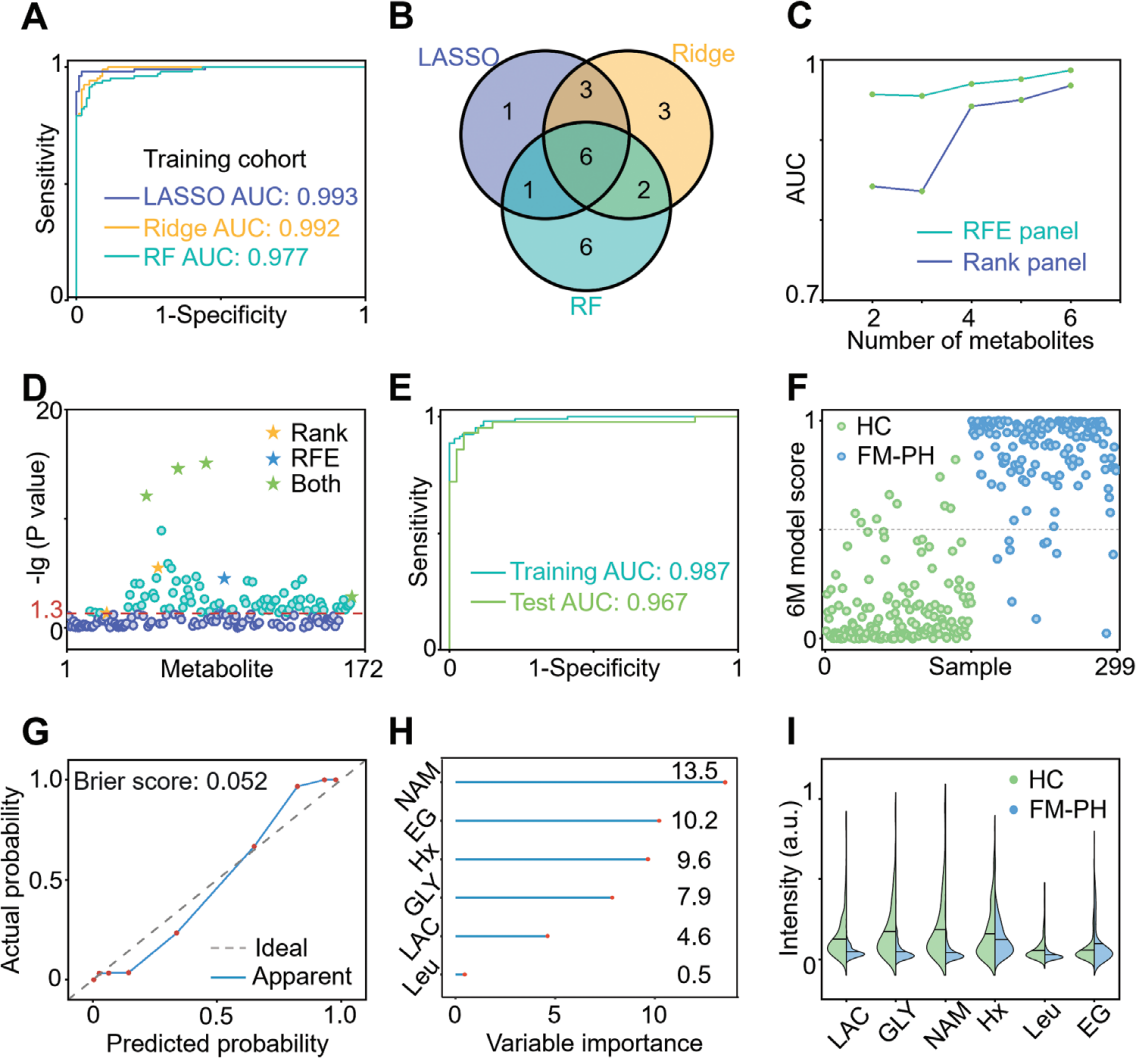
FM‐PH diagnostic model derivation and validation. A) Receiver operator characteristic (ROC) curves of the least absolute shrinkage and selection operator (LASSO), ridge regression (Ridge), and random forest (RF) models built with 172 metabolites. B) Venn diagram of shared metabolites between the top 20 metabolites obtained from the LASSO, Ridge, and RF models. C) Comparison of the areas under the receiver operating curve (AUCs) obtained from the RF model with different metabolite panels selected by recursive feature elimination (RFE) and metabolite ranking. D) The six metabolites selected by RFE and metabolite rank were significantly different between HC and FM‐PH groups. The dashed line referred to the boundary of *p* = 0.05. E) ROC curves, F) sample classification score, and G) calibration curve of the model built with six metabolites selected by RFE (6m model). H) The variable importance of six metabolites in the 6m model. I) Violin diagram for the intensities of six metabolites in the HC group (green) and FM‐PH group (blue).

Machine learning was next used to develop a model for predicting the clinical status. We performed tenfold cross‐validation with all metabolites to construct diagnostic models based on three algorithms (least absolute shrinkage and selection operator (LASSO), ridge regression (Ridge), and random forest (RF). Patient classification (**Figure** **2**A) was achieved, with areas under the receiver operating curve (AUC) of 0.993 (LASSO, (95% confidence interval (CI): 0.984–1, sensitivity of 0.972, specificity of 0.982), 0.992 (Ridge, 95% CI: 0.984–0.999, sensitivity of 0.906, specificity of 0.973), and 0.977 (RF, 95% CI: 0.962–0.993, sensitivity of 0.925, specificity of 0.936). These results indicated that the employment of machine learning highlighted significant metabolic differences between FM‐PH and HC groups, enabling accurate screening for FM‐PH.

Screening for valuable features and eliminating interferences are crucial for discriminant modeling and disease marker discovery. These three algorithms enable the assessment of the importance of each metabolite in patient differentiation. Following specific filter criteria (*p* < 0.05, in the top 20 of the feature rank of all three algorithms), six metabolites were identified as significant (Figure 2B). These metabolites include dihydrothymine, lactate (LAC), succinic acid (SUC), glyceric acid (GLY), niacinamide (NAM), malic acid (MAI), and estriol‐3‐glucuronide (EG).

While each metabolite among the top six demonstrated significant potential for discriminating between FM‐PH and HCs, their collective use may not have yielded optimal diagnostic performance. To identify the most effective diagnostic panel of metabolites that are maximally relevant and minimally redundant, we employed recursive feature elimination (RFE) alongside the RF algorithm in the training cohort. We compared the diagnostic performances of models, with the number of features progressively increasing from two to six, as selected by both the algorithm's rank (Rank panel) and RFE (REF panel, Figure 2C). All models achieved AUCs greater than 0.8. Notably, the AUC of the panels selected by RFE consistently outperformed those derived from simple combinations of top‐ranked metabolites. The RFE process identified a panel of six metabolites—LAC, GLY, NAM, hypoxanthine (Hx), leucine (Leu), and EG—that were significantly different (*p* < 0.05, Figure 2D) between the FM‐PH and HC groups. The model incorporating this panel (referred to as the 6m model) exhibited the highest predictive effectiveness for FM‐PH diagnosis and will be utilized in subsequent experiments. The findings indicated that despite a substantial reduction in metabolic features (from 12 000 signals to six metabolites), accurate differentiation between FM‐PH patients and HCs remains achievable.

We further verified the validity of the 6m model for the diagnosis of FM‐PH. Upon tenfold cross‐validation in the training cohort, the model achieved a class‐weighted average AUC of 0.987 (95% CI: 0.976–0.998, sensitivity of 0.925, specificity of 0.955). In the subsequent validation with the test cohort (Figure 2E), the 6m model yielded an AUC of 0.967 (95% CI: 0.925–1, sensitivity: 0.953, specificity: 0.900). Employing a cutoff value of 0.5, we accurately identified 93.3% of the FM‐PH patients (*n* = 139) and 94.0% of the HC participants (*n* = 141, Figure 2F), yielding an overall accuracy of 93.6%. The calibration curve (Figure 2G) demonstrated strong agreement between predicted and observed results across all samples with a Brier score of 0.052. This indicated the diagnostic capacity of the 6m model in clinical practice, suggesting a Brier score of less than 0.25 which is an appropriate threshold according to previous literature.^[^ ^12^ ^]^

Notably, the contributions of the six metabolites to the 6m model were detailed in Figure 2H and Table S2 (Supporting Information). Significant differences (*p* < 0.05) in the levels of these biomarker candidates between HC and FM‐PH groups are shown in Figure 2I. Among these metabolites, the first five (LAC, GLY, NAM, Hx, and Leu) were downregulated in patients, while the sixth metabolite (EG) was upregulated. These six metabolites, which were implicated in the etiology and energy metabolism of FM‐PH, provided insights for further exploration of the underlying mechanisms of this disease.

### 2.4 PH‐Subtype Discrimination

**Figure 3:**
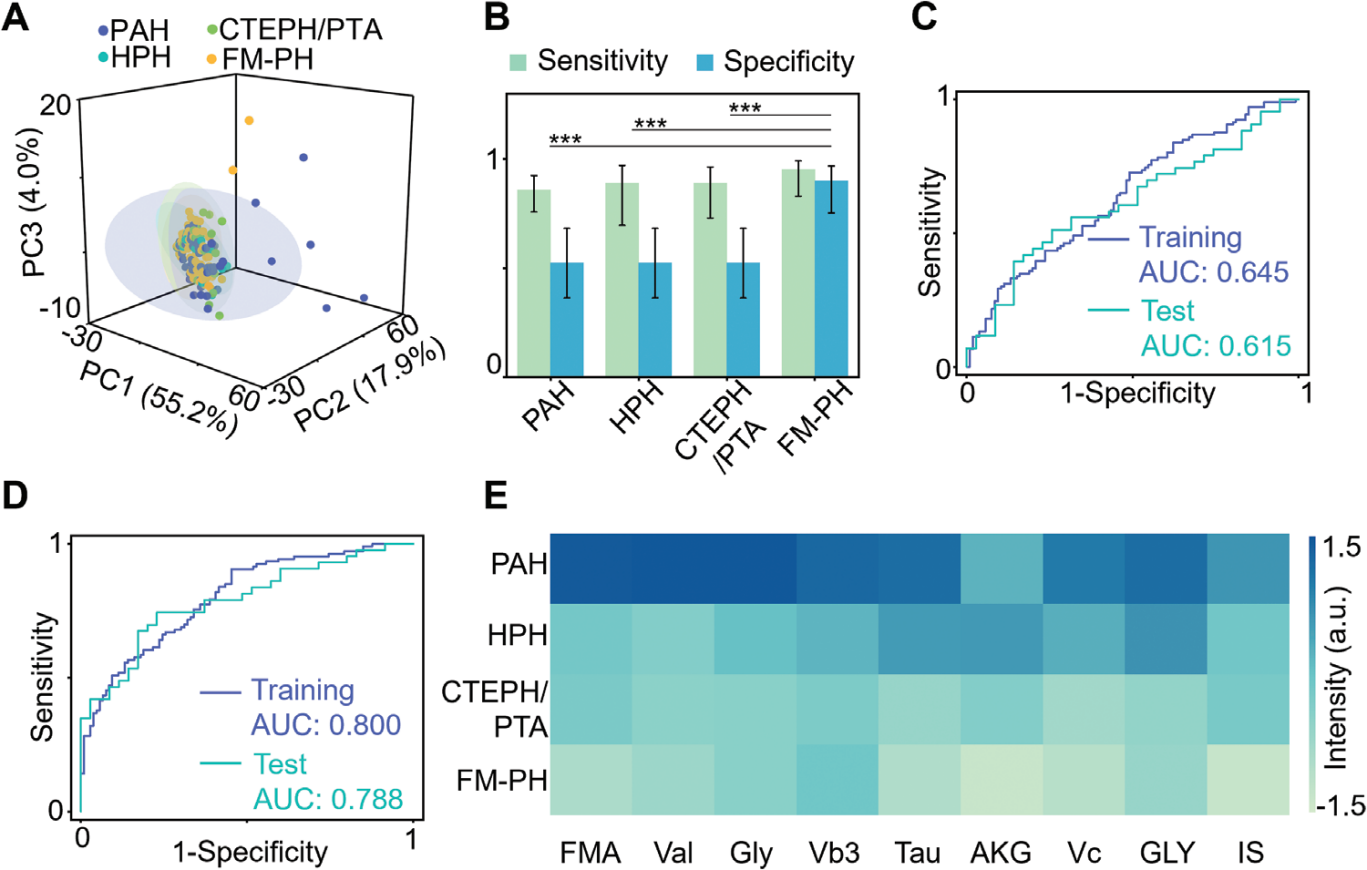
PH‐subtype discrimination. A) Principal component analysis showed a certain degree of overlap among four PH subtypes. B) Diagnostic efficacy of the 6m model in different PH subtypes. ROC curves of the subtyping model built with C) 172 metabolites and D) 8 metabolites selected by RFE and two clinical factors (MF model). Violet referred to ROC curves obtained from 10‐fold cross‐validation for the training cohort; cyan referred to ROC curves obtained for the test cohort. E) A heatmap of nine metabolites selected by feature ranking exhibited significant differences among PH subtypes.

PH is frequently associated with a variety of predisposing factors and is classified into five subtypes according to WHO guidelines. The ability to differentiate between PH subtypes is crucial for clinical dialogue, influencing patient treatment and care.^[^ ^13^ ^]^ However, the different subtypes of PH may exhibit varying degrees of similarity, which can hinder accurate clinical diagnosis. For instance, FM‐PH is often misdiagnosed as CTEPH because of its clinical features with pulmonary vascular stenosis.^[^ ^4^ ^]^ Principal component analysis revealed similar metabolic profiles with subtle differences among patients across four subtypes of PH (**Figure** **3**A). Consequently, we examined the performance of the 6m model in diagnosing other PH subtypes. The 6M model outperformed its diagnostic capabilities for FM‐PH when applied to other PH subtypes (Figure 3B). Importantly, the average specificity decreased by 41.7%, resulting in a high false‐positive rate of 0.475 when using the 6m model for other PH subtypes.

We applied a RF model to investigate the most significant metabolites and their potential for subtyping PH. The RF model, built with all 172 metabolites, achieved an AUC of 0.645 (accuracy: 58.5%) during model training, and 0.615 (accuracy: 57.7%) during testing (Figure 3C). To determine a minimal panel of metabolites that could effectively distinguish patients with FM‐PH from those with other PH subtypes, we further optimized the selection and combination of metabolites using RFE. As shown in Figure S4A (Supporting Information), the model comprising eight metabolites (8M model), consisting of glycine (Gly), fumarate (FMA), creatine, purine, taurine (Tau), 2‐oxoglutarate (AKG), ascorbate (Vc), and Hx, reached an AUC of 0.728. To enhance the effectiveness of FM‐PH subtyping, we integrated metabolite features with several clinical information reported to be the independent predictive factors for FM‐PH subtyping. We combined eight metabolites with tuberculosis (single‐factor AUC of 0.671) and the natural logarithm of *N*‐terminal pro‐B‐type brain natriuretic peptide (lnBNP, single‐factor AUC of 0.631, Figure S4B, Supporting Information). As a result, the FM‐PH subtyping model constructed with eight metabolites and two clinical factors (MF model) achieved FM‐PH distinction from other PH subtypes with an AUC of 0.800 in the training cohort and 0.788 in the test cohort (Figure 3D). The combination of metabolites and clinical factors significantly improved FM‐PH classification compared to models constructed using single factors (*p* < 0.05, Table S3, Supporting Information).

The PH patients received various pharmacological therapies with agents potentially affecting the endothelium. To ensure that the observed metabolic differences between subtypes reflect distinct pathological features rather than variations in treatment strategies, we controlled for the potential confounding effects of medication use (Table S4, Supporting Information). Specifically, we selected patient samples within one of the PH subtypes (PAH) and stratified them into three groups according to their medication regimens: targeted agents alone (*n* = 13), diuretics alone (*n* = 6), and anticoagulants alone (*n* = 3). We performed the analysis of variance to assess the impact of medication on metabolite profiles across these groups. The findings indicated that different pharmacological treatments did not significantly alter the metabolism (*p* > 0.05). Consequently, the observed metabolic perturbations were not influenced by the different pharmacological therapies, but rather by metabolic stimuli associated with the pathology of PH.

While these eight metabolites played important roles in the discrimination for FM‐PH, they did not encapsulate the core differences between FM‐PH and the other subtypes. Exploring metabolic differences among different PH subtypes requires the identification of features that are specifically valuable for each subtype. Using the feature ranking from the RF model, we selected nine metabolites that exhibited significant differences between FM‐PH and other PH patients from the top ten features (Figure 3E). These nine metabolites—FMA, AKG, Vc, indoxyl sulfate (IS), nicotinate (Vb3), valine (Val), Gly, Tau, and GLY—highlighted the distinctions in etiology and pathology among PH subtypes. The higher consumption of these metabolites in FM‐PH and their accumulation in other PH subtypes resulted in their general under‐representation in FM‐PH.

## 3 Discussion

This study analyzes circulating metabolites across different subtypes of PH. It robustly identifies and validates differences between HC and PH groups (e.g., LAC, GLY, NAM, Hx, Leu, and EG). These findings enhance the evidence that these metabolites may serve as diagnostic and subtyping biomarkers.

Warburg effect is a distinctive characteristic of PH, whereby heightened aerobic glycolysis leads to elevated levels of LAC. Conversely, other studies suggest that LAC is utilized by pulmonary cells as an energy source under conditions of inadequate oxygen, fueling the progression of PH.^[^ ^14^ ^]^ Gly and Leu have been reported to be downregulated in tuberculosis patients, which are contributing factors to FM‐PH.^[^ ^15^ ^]^ Abnormal immune proliferative response in the mediastinum in response to *Mycobacterium tuberculosis* results in structure involvement of the FM, further inducing PH. Additionally, disturbances in Gly metabolism were also associated with the reduced expression of BOLA3, contributing to increased endothelial proliferation and vasoconstriction, which collectively drive the progression of PH.^[^ ^16^ ^]^ Over the years, accumulating evidence has supported a direct pathogenic role of uric acid in the pathophysiology of PAH, which may explain the downregulation of its precursor, Hx, observed in this study.^[^ ^17^ ^]^ Purine metabolism has been shown to promote vascular remodeling in preclinical models of PAH.^[^ ^18^ ^]^ NAM, which is part of the mammalian nicotinamide adenine dinucleotide biosynthesis pathway, is associated with dysfunction and injury of pulmonary artery endothelial cells, a hallmark of PH.^[^ ^19^ ^]^ The increased risk of PH in women can be partly attributed to estrogen signaling; correspondingly, we found higher average levels of estrogen catabolite and EG in the FM‐PH group.^[^ ^20^ ^]^ Estrogen also promotes pulmonary vasculature smooth muscle cell hyperproliferation by inhibiting BMPR2 expression, partly explaining why women are more susceptible to PAH.^[^ ^21^ ^]^ The metabolic disorders in FM‐PH are primarily linked to their causative factors and pathological manifestations. This proposition is also supported by the physiological functions of the representative differential metabolites identified in our study.

Moreover, the distinguishing performance between certain subtypes of PH remains suboptimal within the current diagnostic framework, particularly in differentiating FM‐PH from other subtypes. These challenges in subtyping arise from the similarities in distinguishing characteristics among the overlapping groups.^[^ ^13^ ^]^ Emerging approaches to phenotyping increasingly rely on omics‐based data, with metabolic analysis offering significant clinical impact by enabling simultaneous characterization of subtype‐specific information.^[^ ^22^ ^]^

In the context of metabolic differences across various PH subtypes, purine and Hx play contribute to the pathogenesis of PAH, by promoting oxidative stress and enhancing endothelium‐bound xanthine oxidase activity.^[^ ^17^ ^]^ Several researchers have reported that creatine accumulation can improve cellular responses to hypoxic conditions, which may elucidate the relationship between creatine and hypoxia‐related PH.^[^ ^23^ ^]^ FMA and AKG play roles in the citric acid cycle, and their varying levels among different PH subtypes suggest differing degrees of impaired mitochondrial function.^[^ ^24^ ^]^ As vitamins, ascorbate, and nicotinate are essential for antioxidant defense; nicotinate also influences vascular health and enhances muscle function.^[^ ^19^, ^25^ ^]^ Most patients with FM‐PH have a history of tuberculosis, and reduced levels of Val, Gly, Tau, and GLY—metabolites involved in amino acid catabolism—are associated with energy supply to the immune system, as documented in tuberculosis literature.^[^ ^15^, ^26^ ^]^ Additionally, high concentrations of IS in plasma samples from other PH patients are linked to cardiovascular disease,^[^ ^27^ ^]^ which can trigger certain forms of PH. Poor glucose control is another characteristic of mainstream PH.^[^ ^28^ ^]^

Taken together, our platform enables the concurrent gathering of metabolic information, facilitating the diagnosis of FM‐PH and differentiation from other PH subtypes. As a result, the 6m model based on metabolic information distinguishes FM‐PH from HC participants with an AUC of 0.987, thus facilitating accurate diagnosis. The MF model also demonstrates significant capability in differentiating FM‐PH, achieving an AUC of 0.800, which marks an improvement over existing diagnostic methods that are often hampered by high misdiagnosis rates, underscoring the feasibility of our methodology.

While the results demonstrate favorable AUC values, further exploration of the model's feasibility in actual clinical applications is warranted. The current diagnostic process for FM‐PH typically begins with invasive RHC to confirm the presence of PH, followed by various imaging techniques to identify the specific subtype of the disease.^[^ ^5^ ^]^ In contrast, blood sample collection enhances patient safety, benefiting those who may not tolerate lengthy clinical assessments.^[^ ^29^ ^]^ Based on the inherent advantages of FPELDI MS detection, we integrated the model into existing clinical workflows^[^ ^3^ ^]^ and assessed clinicians' acceptance of new diagnostic tools (Figure S5, Supporting Information).

The integration of diagnostic models minimized the need for further unnecessary investigations. In a typical clinical scenario, clinicians can utilize the MF model to analyze plasma metabolic profiles for patients with PH as the chief complaint. Patients testing positive are directly triaged for gold‐standard validation of FM, thereby eliminating the need for three redundant exclusion tests in this subgroup. Conversely, patients testing negative proceed through the standard PH subtyping workflow, which involves a comprehensive diagnostic evaluation. In cases where suspicious symptom‐including unexplained hemoptysis, refractory pleural effusion, superior vena cava syndrome, or unexplained dyspnea‐is the primary clinical manifestation, the 6M model can differentiate FM‐PH from HC individuals. Patients with positive results proceed to undergo gold‐standard confirmation, while those with negative results are immediately excluded from the FM diagnostic pathway, sparing them from the complexities of contrast‐enhanced chest CT. The incorporation of diagnostic models reduces the physical, temporal, and financial burdens on patients associated with the conventional diagnostic process, also promoting the optimal distribution of clinical resources and improving medical service efficiency.

For population health benefits, the promotion strategy of the FPELDI MS platform in real‐world clinical scenarios hinges on the bulk processing of samples, which is facilitated by the scale effect achieved in a centralized laboratory through high‐throughput detection.^[^ ^30^ ^]^ Generally, the equipment costs and operational complexity may impede the clinical application of the FPELDI MS platform. Inspired by next‐generation sequencing technologies (e.g., Illumina NovaSeq 6000), the platform utilizes a 10^6^‐level microarray (such as the BGI DNBSEQ‐T7 chip) to achieve parallel detection of 3000 samples in a single run. Results indicate that when sample throughput increases from 10 to 100, the marginal cost curve exhibits an inflection point, whereby the sequencing cost per sample reduces by 90% (based on actual operational data from the Stanford Genome Center in 2023). Similarly, FPELDI MS showcases substantial throughput advantages, processing at least 384 samples in a single run, with each sample requiring merely a few seconds for analysis. Thus, a scaled batch detection strategy facilitates cost reduction and operational simplification of the proposed platform, ultimately enhancing its clinical applications in healthcare settings.

However, several limitations must be addressed: first, this research was conducted at a single center, and the diagnostic performance should be further validated by incorporating larger sample sizes from multiple centers. Second, the limited sample size of PH subtypes other than FM‐PH restricts the exploration of metabolic differences among these subtypes. Therefore, future research should focus on optimizing the subtyping model using cohorts with a broader range of PH subtypes that exhibit overlapping characteristics. Moreover, we did not explore the information contained in the disturbed metabolites related to patient survival rates and treatment outcomes,^[^ ^31^ ^]^ which could be utilized for precise prognosis and precision intervention. Investigating the relationship between metabolites and patient prognosis in future studies holds significant clinical value. These efforts will enhance diagnostic precision and personalized treatment guidance, while also providing a deeper understanding of the biochemical differences that distinguish various PH subtypes.

Collectively, we revealed metabolic disorders in PH and incorporated machine learning algorithms to construct two models that identify FM‐PH patients and distinguish them from other PH subtypes, respectively. Our work demonstrated FM‐PH diagnosis using a single detection and enhanced the understanding of PH pathology.

## Conflict of Interest

The authors declare no conflict of interest.

## Supporting information



Supporting Information

## Data Availability

The data that support the findings of this study are available from the corresponding author upon reasonable request.
