# Supplementary material for: Single Test‐Based Diagnosis and Subtyping of Pulmonary Hypertension Caused by Fibrosing Mediastinitis Using Plasma Metabolic Analysis
Source: Adv Sci (Weinh). 2025 Mar 6;12(17):2416454. doi: 10.1002/advs.202416454 (PMC12061239; doi:10.1002/advs.202416454)
Supplement: Supplementary file 1 — Supporting Information [file ADVS-12-2416454-s001.docx]

Supporting Information

Single test-based diagnosis and subtyping of pulmonary hypertension caused by fibrosing mediastinitis using plasma metabolic analysis

Yating Zhao†, Chunmeng Ding†, Hongling Su, Aqian Wang, Aiping Tang, Hongfan Zhao, Ya Ma, Min Zhang, Wanshan Liu, Ruimin Wang, Ziyue Zhang, Shouzhi Yang, Dingyitai Liang, Yida Huang, Kun Qian, Lin Huang*, Qihua Fu*, and Yunshan Cao*

This section includes the following:

1. Experimental Section/Methods

2. Figure S1-5

3. Table S1-4

**Experimental Section/Methods**

*Materials and reagents*

This work included reagents for the preparation of ferric particles and mass spectrometry analysis. Ferric chloride hexahydrate (FeCl_3_·6H_2_O, 99.0%), trisodium citrate dihydrate (Na_3_C_6_H_5_O_7_·2H_2_O, 99.5%), ethylene glycol (C_2_H_6_O_2,_ 99.5%), and ethanol absolute (C_2_H_6_O, 100%) were procured from Sinopharm Chemical Reagent (Shanghai, China). Sodium acetate anhydrous (NaC_2_H_3_O_2_, 99.0%) was purchased from Aladdin Reagent (Shanghai, China). Methanol (CH_4_O, HPLC) and acetonitrile (C_2_H_3_N, HPLC) were purchased from Sigma-Aldrich (St. Louis, MO, USA).

*Synthesis of ferric particles*

We present an improved solvothermal method for the synthesis of ferric particles. Briefly, 0.6g trisodium citrate dihydrate and 2.4g ferric chloride hexahydrate were dissolved successively in 100 mL of ethylene glycol and dispersed by ultrasound at room temperature for 90 minutes to ensure complete dissolution. Subsequently, sodium acetate anhydrous was added to the solution, and the mixture was stirred for 45 minutes. The solution system was then transferred to a Teflon-lined autoclave and heated at 200°C for 10 hours. After the reaction, the autoclave was allowed to cool to room temperature. The product was collected by centrifugation and washed with ethanol and distilled water to remove impurities. The final product was dried under vacuum at 60°C for 12 hours. The morphology of ferric particles was characterized by scanning electron microscope (SEM) using Gemini 300 (ZEISS, Germany). Nano Measurer (version 1.2) was applied to measure the size distribution of ferric particles in the SEM result. 3D confocal reconstruction images revealed the crystallization status of ferric particles using a KEYENCE VK-X3000 (KEYENCE, Japan).

*Human plasma samples*

The Medical Ethics Committee of Sichuan Provincial People's Hospital approved this study (approval 2024-470). We obtained written informed consent from all participants who underwent blood collection. This study recruited 150 healthy control (HC) and 303 pulmonary hypertension (PH) participants. Inclusion criteria of patients include (1) patients who received right heart catheterization and mean pulmonary artery pressure (mPAP) < 20 mmHg and (2) samples without hemolysis during the sample handling. (Figure S1). All plasma samples included (n=440) were stored at -80°C.

*Mass spectrometry (MS) analysis*

Ferric particles were dispersed to 1 mg/mL in water to obtain matrix suspensions. Before analysis, plasma samples were subjected to a protein precipitation process utilizing a methanol-acetonitrile (1:1, v/v) solution. 50 μL plasma was mixed with an equal volume of the methanol-acetonitrile solution. The mixture was vortexed for 1-2 minutes to ensure thorough mixing and then incubated at -20°C for a minimum of 30 minutes to facilitate deproteinization. The mixture was then centrifuged at 10000 rpm for 15 minutes. The supernatant was carefully collected and transferred to a clean tube for subsequent analysis.

During MS analysis, 1 μL of deproteinized plasma was dropped onto the target plate and dried naturally. Then, 1 μL of matrix suspension was added to the sample point and dried at room temperature. We used autoflex maX MALDI-TOF MS (Bruker, Germany) instruments with a 355 nm Nd:YAG laser to detect the samples on the target plate. Mass spectra were acquired in positive ion reflector mode. The test program parameters are configured as follows: positive ion reflector mode, 60% laser intensity, acceleration voltage of 20 KV, and repetition rate of 200 Hz. All MS results were exported directly for further analysis.

*Data processing*

The data processing was carried out using Python (version 3.8). Baseline correction and spectral smoothing were performed to eliminate noise in raw mass spectra. Peaks with signal-to-noise greater than 3 and present in ≥2/3 of the samples were extracted from each sample for subsequent analyses.

In the model-building phase, all extracted peak information was utilized to construct several predictive models, by using LASSO, Ridge, and Random Forest algorithms. Each model was developed to assess the importance of different features in classifying effectively. Recursive feature elimination (RFE) was also employed to refine the metabolite panel, systematically removing less significant peaks to identify the optimal combination of features for reliable classification. The final selected metabolites were identified using the Human Metabolome Database and incorporated into the classification model for effectiveness verification. All the models were constructed by Orange (version 3.36.2). RFE was realized using SPSSPRO (version 1.1.28, https://www.spsspro.com/).

Mann–Whitney U tests, unpaired t-tests, and analysis of variance (ANOVA) were conducted using IBM SPSS Statistics (version 24). Data with *P* < 0.05 were considered as statistically significant.

**
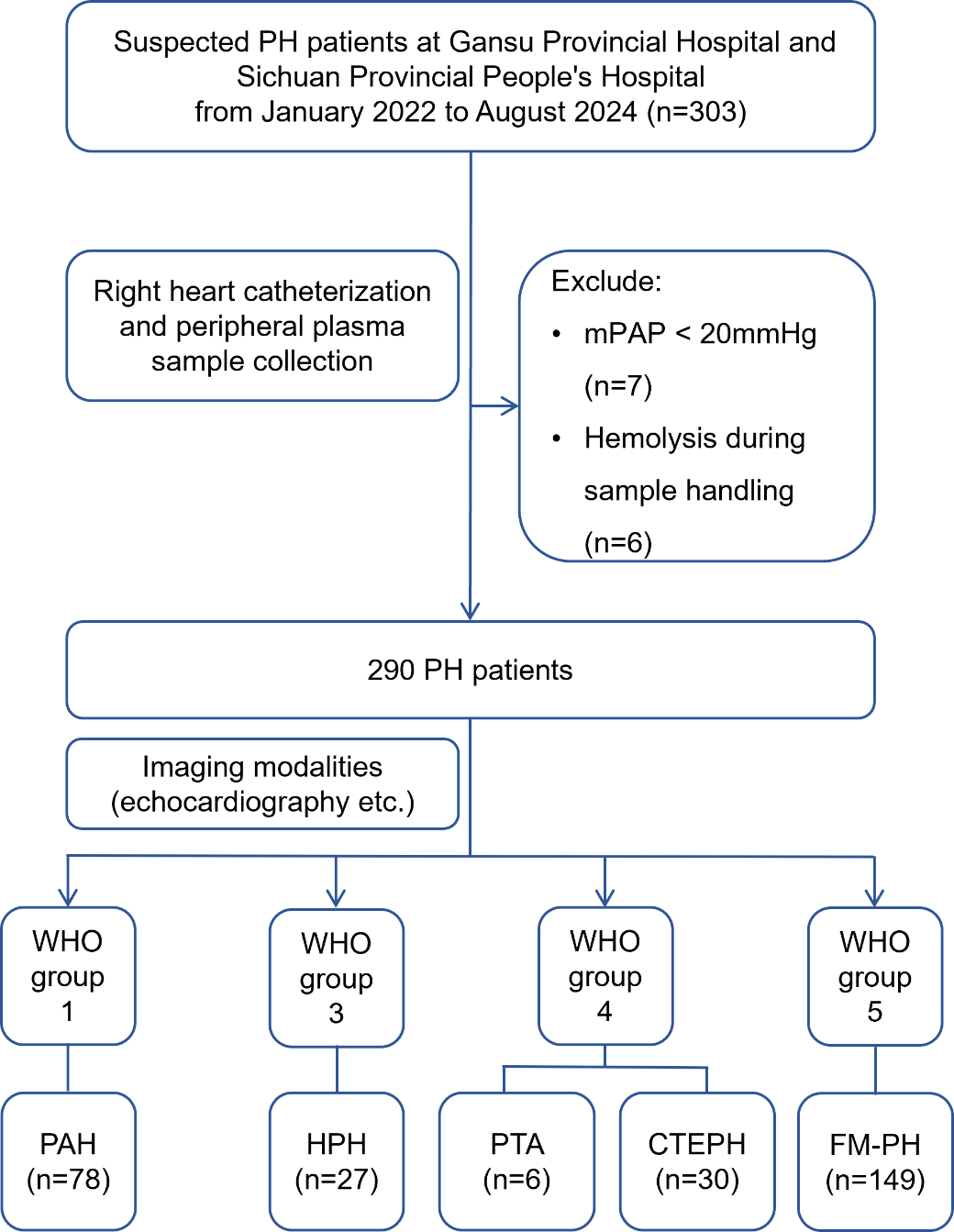
**

**Figure S1. Inclusion-exclusion cascade of patients.** Abbreviates: mPAP: mean pulmonary artery pressure; PH: pulmonary hypertension; PAH: pulmonary arterial hypertension; HPH: hypoxic-associated pulmonary hypertension; CTEPH: pulmonary hypertension related to chronic thromboembolic disease; PTA: pulmonary Takayasu arteritis; FM-PH: PH caused by fibrosing mediastinitis.

**
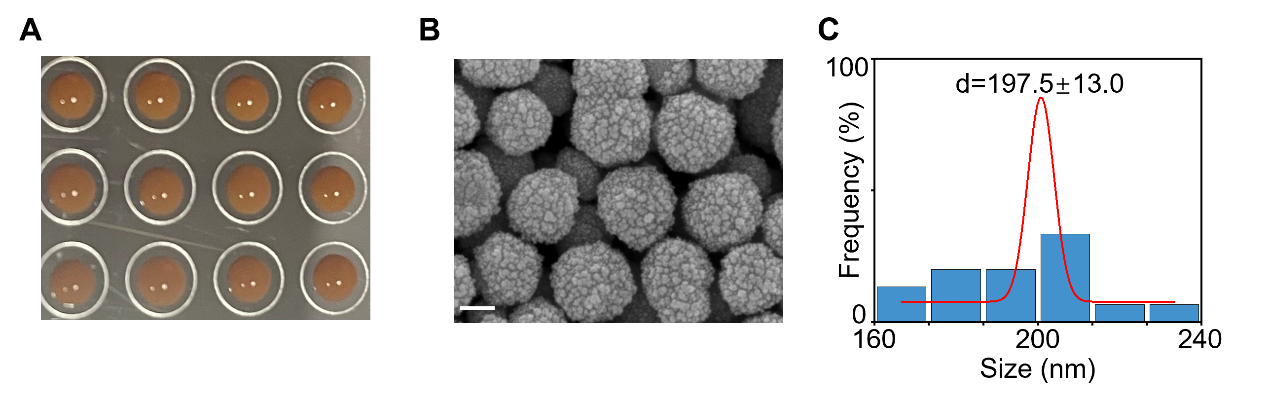
**

**Figure S2. Characterization of ferric particles.** A) Digital image shows the target plate with loaded ferric particles. B) Scanning electron microscope (SEM) images exhibited the nanoscale rough surface of the ferric particles. The scale bar was 100 nm. C) The particle size distribution histogram of the ferric particles indicated a size of 197.5 ± 13.0 nm.


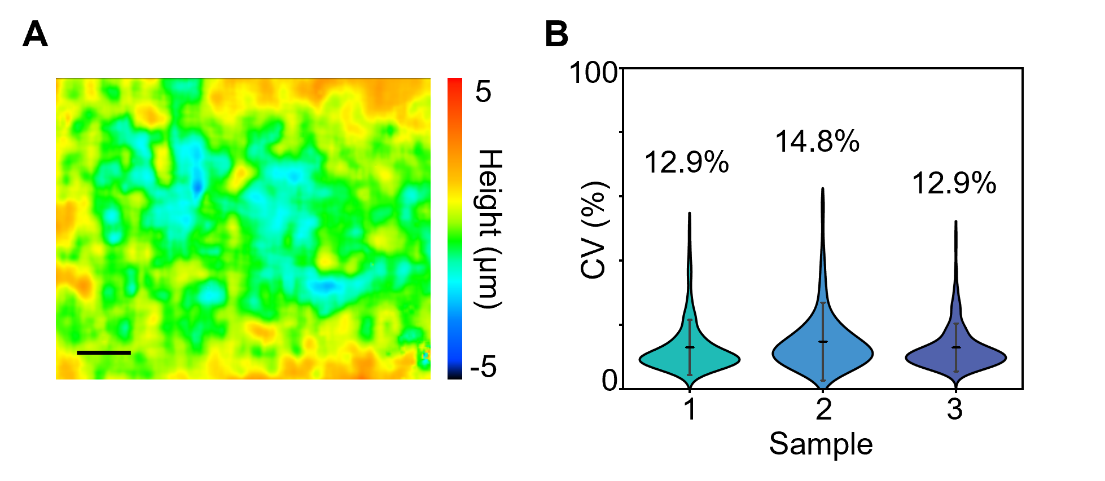


**Figure S3. Reproducibility of ferric particle-enhanced laser desorption/ionization mass spectrometry analysis.** A) Corresponding 3D reconstruction images of matrix crystallization. The scale bar was 100 nm. B) Coefficients of variation (CV) of 10 independent replicates were acquired across three plasma samples.


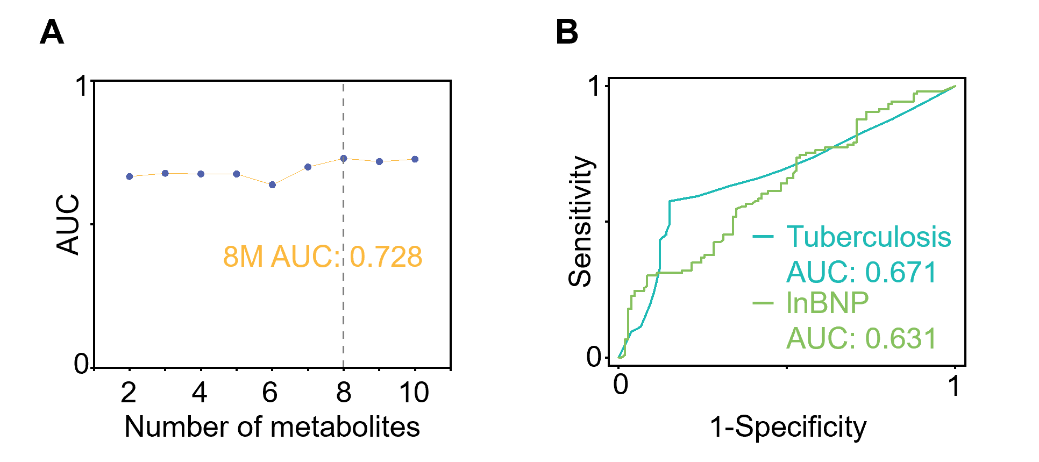


**Figure S4.** **FM-PH subtyping results.** Areas under the receiver operating curve (AUCs) were obtained from subtyping models constructed with A) different metabolite panels selected by recursive feature elimination. The dashed line referred to the optimal AUC acquired from the panel consisting of eight metabolites (8M model). B) The receiver operating (ROC) curves for the subtyping model designed to distinguish between FM-PH and other PH subtypes. Cyan referred to the ROC curve obtained by a model constructed with information on the presence of tuberculosis; green referred to the ROC curve obtained from a model built with the natural logarithm of N-terminal pro-B-type brain natriuretic peptide (lnBNP).


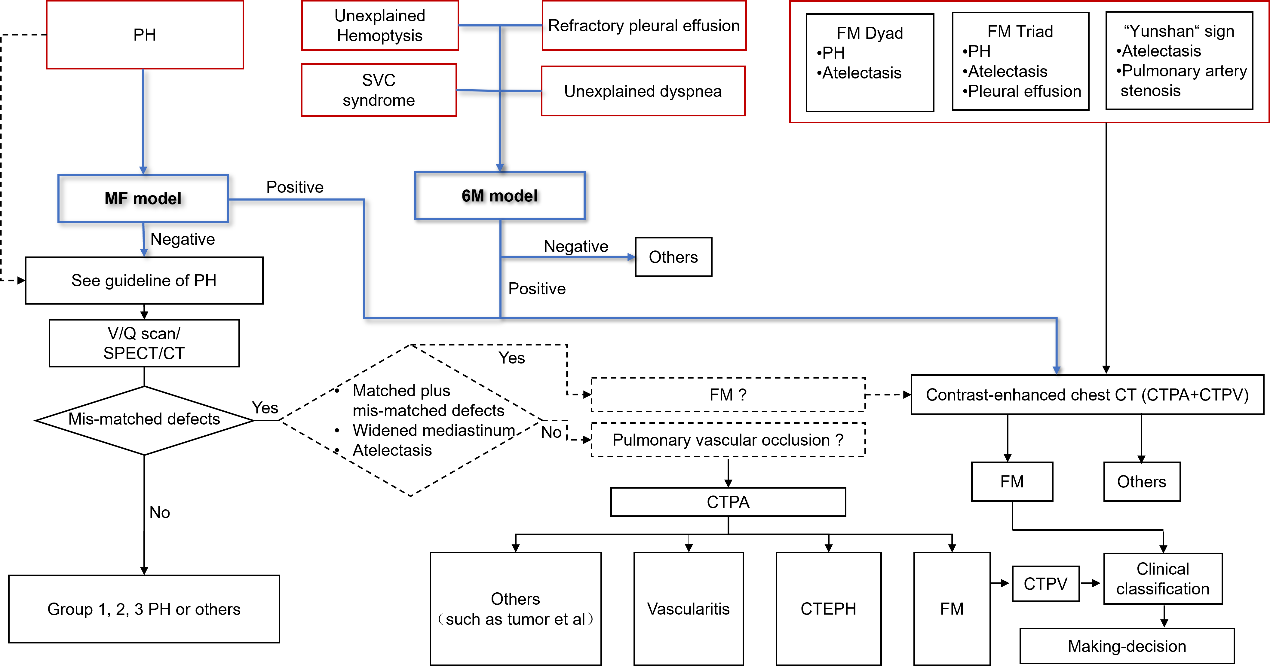


**Figure S5. Diagnostic workflow of FM-PH integrated diagnostic models.** Red frames refer to the chief complaint of patients; blue frames refer to our proposed diagnostic models; dotted-line frames refer to steps could be omitted after integrated our models into the clinical workflow. MF model: PH subtype-distinguish model; 6M model: FM-PH diagnostic model; CT = computed tomography; CTEPH = chronic thromboembolic pulmonary hypertension; CTPA = computed tomography pulmonary angiography; CTPV = computed tomography pulmonary venography; FM = fibrosing mediastinitis; PH = pulmonary hypertension; PH-FM = pulmonary hypertension caused by fibrosing mediastinitis; SPECT/CT = single-photon emission computed tomography/CT; SVC = superior vena cava; V/Q = lung ventilation/perfusion.

**Table S1.** Clinical characteristics of patients.

| Characteristics | PAH  (n=78) | HPH  (n=27) | CTEPH/PTA  (n=36) | FM-PH  (n=149) | *P* value |
| --- | --- | --- | --- | --- | --- |
| Age, years | 44.38±1.80 | 60.70±2.17^a)^ | 54.58±2.01^b, d)^ | 66.70±0.57^c, e, f, g)^ | < 0.001 |
| Gender, n (%) |  |  |  |  | < 0.001 |
| Male | 18 (23.08) | 12 (44.44) | 9 (25.00) | 59 (39.60) |  |
| Female | 60 (76.92) | 15 (55.56) | 27 (75.00) | 90 (60.40) |  |
| Height, cm | 160.85±1.01 | 162.15±1.36 | 162.36±1.29 | 162.69±0.62 | 0.418 |
| Weight, kg | 58.13±1.31 | 59.00±2.18 | 63.58±2.05 | 58.97±0.86 | 0.098 |
| BMI, kg/m² | 22.60±0.60 | 39.63±17.24^a)^ | 24.10±0.66^d)^ | 22.28±0.31^e)^ | 0.024 |
| Vital signs | N/A |  |  |  |  |
| T, ℃ | 36.26±1.18 | 36.43±0.18 | 36.36±0.19 | 36.42±0.22 | 0.330 |
| HR, bpm | 85.64±14.61 | 90.30±15.98 | 78.11±10.52^b,d)^ | 84.91±12.71^f)^ | 0.003 |
| RR, bpm | 19.55±1.53 | 19.89±0.97 | 19.97±1.38 | 19.74±1.39 | 0.437 |
| SBP, mmHg | 111.10±18.96 | 118.44±18.96 | 117.25±13.58 | 122.46±17.76^c)^ | <0.001 |
| DBP, mmHg | 72.49±12.33 | 79.07±15.73^a)^ | 76.72±10.50 | 77.28±11.11^c)^ | 0.016 |
| Risk stratification |  | (n=26) |  | (n=142) | <0.001 |
| Low-risk | 26 (33.33) | 6 (23.08) | 16 (44.44) | 62 (43.66) |  |
| Intermediate-risk | 29 (37.18) | 10 (38.46) | 20 (55.56) | 58 (40.85) |  |
| High-risk | 23 (29.49) | 10 (38.46) | 0 (0) | 22 (15.49) |  |
| WHO FC, n (%) |  | (n=25) |  |  | 0.169 |
| Ⅰ | 2 (2.56) | 0 (0) | 0 (0) | 2 (1.34) |  |
| Ⅱ | 43 (55.13) | 6 (24.00) | 20 (55.56) | 67 (44.97) |  |
| Ⅲ | 29 (37.18) | 17 (68.00) | 15 (41.67) | 74 (49.66) |  |
| Ⅳ | 4 (5.13) | 2 (8.00) | 1 (2.77) | 6 (4.03) |  |
| 6MWD, m | 386.21±9.52  (n=61) | 338.17±20.54  (n=12) | 348.00±18.88  (n=30) | 323.33±8.98^c)^  (n=103) | < 0.001 |
| ≤ 165 | 1 (1.64) | 0 (0) | 2 (6.67) | 4 (3.88) |  |
| ＞ 165 | 60 (98.36) | 12 (100.00) | 28 (93.33) | 99 (96.12) |  |
| NT-proBNP, ng/L | 1770.43±253.76  (n=76) | 1632.74±501.10  (n=25) | 1553.28±361.57  (n=36) | 1284.08±303.55  (n=147) | 0.717 |
| ≤ 1100 | 43 (56.78) | 15 (60.00) | 22 (61.11) | 121 (82.31) |  |
| ＞ 1100 | 33 (43.42) | 10 (40.00) | 14 (38.89) | 26 (17.69) |  |
| RHC |  |  |  |  |  |
| sPAP, mmHg | 80.41±3.46 | 52.88±3.67^a)^  (n=25) | 72.47±4.08^d)^ | 59.09±2.06^c, f)^  (n=146) | 0.002 |
| dPAP, mmHg | 37.26±1.92 | 23.76±1.96^a)^  (n=25) | 28.58±1.58^b)^ | 23.36±0.82^c, f)^  (n=146) | < 0.001 |
| mPAP, mmHg | 52.13±2.35 | 33.60±2.40^a)^  (n=25) | 43.00±2.16^b, d)^ | 35.23±1.16^c, f)^  (n=146) | < 0.001 |
| PCWP, mmHg | 6.94±0.30 | 7.15±0.37  (n=26) | 7.00±0.47  (n=35) | 9.14±0.81  (n=145) | 0.099 |
| PVR, WU | 12.70±1.06  (n=77) | 6.37±0.78^a)^  (n=26) | 9.45±0.90^b)^  (n=35) | 7.36±0.35^c)^  (n=147) | < 0.001 |
| CO, L/min | 4.44±0.17 | 4.78±0.25  (n=26) | 4.31±0.26  (n=35) | 4.60±0.10  (n=146) | 0.445 |
| CI, L/min·m^-2^ | 2.80±0.09 | 2.92±0.16  (n=26) | 2.67±0.14  (n=35) | 2.90±0.07  (n=147) | 0.403 |
| SvO_2,_ (%) | 65.08±1.19  (n=77) | 62.81±1.57  (n=26) | 64.06±2.20  (n=35) | 65.82±0.68  (n=147) | 0.432 |
| Echocardiography |  |  |  |  |  |
| PASP, mmHg | 79.91±3.70  (n=75) | 59.19±3.65^a)^  (n=26) | 71.53±4.79  (n=34) | 59.77±2.09^c, f)^  (n=145) | < 0.001 |
| TRV, m/s | 3.98±0.12  (n=69) | 3.68±0.14  (n=26) | 4.04±0.17  (n=34) | 3.62±0.07^c, f)^  (n=145) | 0.012 |
| TAPSE, mm | 19.94±0.77  (n=73) | 17.94±1.14  (n=22) | 21.37±3.11  (n=33) | 20.11±0.54  (n=140) | 0.552 |
| Comorbidity, n (%) |  |  |  |  |  |
| Hypertension | 11 (14.10) | 10 (37.04) | 6 (16.67) | 50 (33.56) | 0.006 |
| Diabetes | 5 (6,41) | 0 (0) | 3 (8.33) | 24 (16.11) | 0.045 |
| COPD | 5 (6.41) | 19 (70.37) | 5 (13.89) | 109 (73.15) | < 0.001 |
| Coronary heart disease | 2 (2.56) | 2 (7.41) | 1 (2.78) | 10 (6.71) | 0.224 |
| Congenital heart disease | 35 (44.87) | 4 (14.81) | 2 (5.56) | 2 (1.34) | < 0.001 |
| Tuberculosis | 0 (0) | 6 (22.22) | 4 (11.11) | 61 (40.94) | < 0.001 |
| Arrhythmia | 40 (51.28) | 11 (40.74) | 18 (50.00) | 49 (32.89) | 0.034 |
| Hyperlipidaemia | 46 (58.97) | 11 (40.74) | 17 (47.22) | 86 (57.72) | 0.257 |
| Hyperuricemia | 30 (38.46) | 7 (25.93) | 14 (38.89) | 43 (28.86) | 0.176 |
| Medications, n (%) |  |  |  |  |  |
| Targeted agent | 60 (76.92) | 11 (40.74) | 18 (50.00) | 2 (1.34) | <0.001 |
| Diuretic | 44 (56.41) | 21 (77.78) | 22 (61.11) | 85 (57.05) | 0.542 |
| Anticoagulant | 16 (20.51) | 9 (33.33) | 33 (91.67) | 54 (36.24) | <0.001 |

**Abbreviates:** PAH: pulmonary arterial hypertension; HPH: hypoxic-associated pulmonary hypertension; CTEPH/PTA: pulmonary hypertension related to chronic thromboembolic disease and pulmonary Takayasu arteritis; FM-PH: PH caused by fibrosing mediastinitis; BMI: body mass index; WHO FC: World Health Organization functional class; 6MWD: 6-minute walking distance; NT-proBNP: N-terminal brain natriuretic peptide; RHC: right heart catheterization; sPAP: systolic pulmonary artery pressure; dPAP: diastolic pulmonary artery pressure; mPAP: mean pulmonary artery pressure; PCWP: pulmonary capillary wedge pressure; PVR: pulmonary vascular resistance; CO: cardiac output; CI: cardiac index; SvO2: mixed venous oxygen saturation; PASP: pulmonary artery systolic pressure; TRV: maximum tricuspid regurgitation velocity; TAPSE: tricuspid annular plane systolic excursion; COPD: chronic obstructive pulmonary disease.

^a)^ *P*<0.05 in PAH and HPH;

^b)^ *P*<0.05 in PAH and CTEPH/PTA;

^c)^ *P*<0.05 in PAH and FM-PH;

^d)^ *P*<0.05 in HPH and CTEPH/PTA;

^e)^ *P*<0.05 in HPH and FM-PH;

^f)^ *P*<0.05 in CTEPH/PTA and FM-PH.

^g)^ Data are presented as mean ± SD or interquartile range unless otherwise stated.

**Table S2.** Metabolite characteristics of the 6M model.

| **Metabolite** | **P value** | **Variable importance** |
| --- | --- | --- |
| Niacinamide | 8.56768E-13 | 13.5 |
| Estriol-3-glucuronide | 0.001441031 | 10.2 |
| Hypoxanthine | 0.031245654 | 9.6 |
| Glyceric acid | 2.79573E-15 | 7.9 |
| Lactic acid | 8.56768E-13 | 4.6 |
| Leucine | 3.21679E-05 | 0.5 |

**Table S3.** Effect of FM-PH subtyping model constructed with different factors.

| **Model** | **AUC** | **95% confidence interval** | ***P* value^a)^** |
| --- | --- | --- | --- |
| MF | 0.800 | 0.741-0.858 | - |
| 8M | 0.728 | 0.660-0.796 | 0.026 |
| tuberculosis | 0.671 | 0.597-0.745 | 9.5×10^-5^ |
| lnBNP | 0.631 | 0.557-0.705 | 1.62×10^-4^ |

**Abbreviates:** AUC: areas under the receiver operating curve; MF: model constructed with eight metabolites and two clinical factors; 8M: model constructed with eight metabolites; tuberculosis: model constructed with information on the presence of tuberculosis; lnBNP: model constructed with the natural logarithm of N-terminal pro-B-type brain natriuretic peptide.

1. Significance of differences in comparison with MF model by DeLong test.

**Table S4.** Analysis of variance.

| Metabolite | F value ^a)^ | P value ^a)^ |
| --- | --- | --- |
| Glycine | 1.45 | 0.26 |
| Fumarate | 1.52 | 0.24 |
| Creatine | 2.16 | 0.14 |
| Purine | 2.46 | 0.11 |
| Taurine | 1.26 | 0.31 |
| 2-oxoglutarate | 0.64 | 0.54 |
| Ascorbate | 0.49 | 0.62 |
| Hypoxanthine | 1.08 | 0.36 |

^a)^ Dependent variable: different medication treatments.
